# Impact of PSP Technique on Clinical Outcomes Following Bioresorbable Scaffolds Implantation

**DOI:** 10.3390/jcm7020027

**Published:** 2018-02-06

**Authors:** Luis Ortega-Paz, Salvatore Brugaletta, Manel Sabaté

**Affiliations:** Department of Cardiology, Cardiovascular Institute, Hospital Clinic, Biomedical Investigation Institute, IDIBAPS, 08036 Barcelona, Spain; lgortega@clinic.cat (L.O.-P.); masabate@clinic.cat (M.S.)

**Keywords:** bioresorbable scaffolds, bioresorbable vascular scaffolds, prognosis, PSP technique, scaffold thrombosis, dual-antiplatelet therapy

## Abstract

Bioresorbable scaffolds (BRS) were introduced in clinical practice to overcome the long-term limitations of newer-generation drug-eluting stents. Despite some initial promising results of the Absorb BRS, safety concerns have led to the discontinuation of the commercialization of this device. Several retrospective studies have assessed the impact of the so-called Pre-dilation, Sizing and Post-dilation (PSP) technique concluding that an optimal PSP technique can improve clinical outcomes following BRS implantation. In this article, the definition of the PSP technique, and the current evidence of its impact on clinical outcomes are put in perspective. Additionality, the relationship between the PSP technique and the dual-antiplatelet therapy to prevent scaffold thrombosis is addressed. Finally, the future perspectives of BRS technology in clinical practice are commented.

## 1. Introduction

Currently, Bioresorbable scaffolds (BRS) are a first-generation technology introduced in clinical practice to overcome the limitations of the newer generation drug-eluting stents (DES) [1,2]. However, this promising technology has suffered several negative results in large randomized clinical trials that have slowed its development [3,4,5]. Specifically, Absorb BRS was associated with a higher rate of target-vessel myocardial infarction and scaffold thrombosis when compared with second-generation DES [6]. Finally, these safety issues led the manufacturer to stop the production of the Absorb BRS.

Several studies have shown that the optimization of the implantation technique can reduce adverse cardiac events [7,8,9,10,11]. Due to the special characteristics of the BRS such as polymeric material, strut thickness, and expansion limits, this technology demands a specific implantation technique that can differ from the metallic stents [12]. Moreover, dual-antiplatelet therapy (DAPT) cessation had raised as an important predictor of scaffold thrombosis [13]. Due to the specific characteristics of BRS such as bioresorption and disturbance of shear stress distribution, it is probable that specific DAPT regimens are needed [14,15].

This review aims to assess the impact of the PSP technique and the DAPT regimens on the clinical outcomes of the patients treated with BRS.

## 2. PSP Technique Concept

PSP is the acronym of the recommended optimal implantation technique according to the manufacturer [16]. The PSP concept has been exclusively studied in the context of the Absorb BRS technology.

Initially, the PSP concept was not clearly defined, and it was integrated within the concept of the five golden ‘P’s: prepare the lesion, properly size, pay attention to the expansion limits, post-dilate with a non-compliant (NC) balloon and pay attention to DAPT [17]. In May 2015, a group of European experts published a consensus document in which they included recommendations regarding the optimal implantation technique, strengthening the concept that BRS should be implanted following a specific protocol [12]. Later in 2016, these recommendations were supported by findings of the MICAT registry (The Coronary Slow-flow and Microvascular Diseases Registry) in which optimization of the implantation technique was associated with a significant reduction in the rate of scaffold thrombosis [7]. In 2017, a post-hoc analysis of the GHOST-EU registry, extended these findings showing a reduction of the device-oriented composite endpoint (DoCE) at 1-year follow-up when all three steps of the PSP technique were performed correctly [8]. Finally, a pooled analysis of the ABSORB trials (ABSORB II, III, CHINA, JAPAN, and EXTEND) found that an optimal PSP technique was strongly associated with clinical outcomes during 3-year follow-up [10].

The Pre-dilation, Sizing, and Post-dilation (PSP) score is a simple scoring model proposed to evaluate the quality of the BRS implantation technique in routine clinical practice. Briefly, it assesses the three critical steps of the BRS implantation technique summarizing it in an individual score. A maximum PSP score has been related to a reduction in 1-year DoCE and a very-high negative predictive value for adverse cardiac events [8]. This score was externally validated in the REPARA registry (2230 patients), obtaining very similar results at 1-year follow-up [18].

The definitions of the PSP technique derived from the GHOST-EU registry are (Table 1):

- Pre-dilation: using an NC balloon 1:1 ratio with reference vessel diameter (RVD) is recommended. If the balloon is not completely expanded, alternative scoring or cutting of balloon should be considered. Eventually, if an optimal lesion preparation is not obtained, a metallic stent should be implanted.
- Scaffold sizing: due to the expansion limits and limited BRS sizes available, to perform an accurate scaffold sizing is critical. The manufacturer has published the recommendations to select the scaffold diameter according to the RVD [16].
  ○ A 2.5 mm diameter scaffold in a vessel with a proximal/distal RVD ≥2.5 mm and <2.75 mm;
  ○ A 3.0 mm diameter scaffold in a vessel with a proximal/distal RVD ≥2.75 mm and <3.25 mm;
  ○ A 3.5 mm diameter scaffold in a vessel with a proximal/distal RVD ≥3.25 mm and ≤3.75 mm;
  ○ If the proximal and distal RVD differed, the mean value is used.
- Post-dilation: using an NC balloon >1:1 ratio with reference vessel diameter up to 0.5 mm (avoid over-expansion) at ≥16 atmosphere is recommended.

## 3. PSP Technique and Intravascular Imaging

Regarding the use of intravascular imaging within the PSP technique, it is not mandatory and mostly it is an angio-guided approach. Angiography guidance and online quantitative coronary angiography (QCA) are used in the three steps of implantation. The importance of the correct estimation of the RVD after a properly pre-dilation should be highlighted, given the fact that the selection of the scaffold diameter and the post-dilation balloon diameter are based on this measurement.

The angio-guided PSP technique has several limitations such as limited information of the atherosclerotic plaque composition, limited visibility of the scaffold in the angiography, difficulties in the estimation of RVD, and uncertainty of a possible scaffold underexpansion or malapposition. However, few studies are comparing intravascular imaging guidance vs. angiography guidance, for the optimization of BRS implantation [25]. A comparison between angio-guided and OCT-guided approaches is shown in Table 1.

Tanaka et al. [9] reported the outcomes of 264 patients treated with BRS in which an optimized implantation technique was applied, and a high rate of intravascular imaging was performed (Intravascular ultrasound 84% and Optical Coherence Tomography 14%). The population was composed mainly of patients with a complex coronary disease with 75% of B2/C type lesion. Operators reported a low threshold for baseline intravascular imaging to confirm vessel diameter and exclude underexpansion or malapposition. The Angiography vs. Intravascular ultrasound Optimisation (AVIO) criteria were used to guide target scaffold area [26]. Interestingly, despite a dedicated implantation protocol, in almost 25% of the cases the use of intracoronary imaging led to further intervention. The main reasons for additional interventions were: underexpansion 21%, malapposition 3%, and edge dissection 1%. Meanwhile, in the patient treated with intravascular imaging guidance, post-dilation balloon/scaffold ratio was higher and final residual percentage stenosis was lower when compared to those treated with an angio-guided approach.

The use of intravascular imaging may be reasonable at least in selected patients [9]. Operators should have a low threshold for intravascular imaging if they have few experience with this technology or if there are any doubts regarding the vessel sizing or post-PCI result.

## 4. PSP Technique and Clinical Outcomes

We will focus on the analysis from the GHOST-EU registry [8] and the ABSORB trials [10]. Table 2 shows a comparison between the two studies. Briefly, these studies differ substantially in their design, enrolled population and methodology. The GHOST-EU data resemble a “real-world” clinical practice including consecutive patients suitable for BRS with limited exclusion criteria. Meanwhile, ABSORB trials are randomized clinical trials with highly selected study population, but with high-quality methods such as core lab analysis, event adjudication, and long-term follow-up. Additionally, it should be highlighted that the definition of optimal PSP technique was assessed differently in each trial. Specifically, PSP technique derived from the GHOST-EU registry defined optimal BRS sizing according to the manufacturer recommendations (see PSP concept section), while the ABSORB trial investigators defined optimal BRS sizing if QCA-RVD was between ≥2.25 mm and ≤3.75 mm (protocol definition). In addition, regarding post-dilation, there is a slight difference in the cutoff of post-dilation pressure (GHOST-EU registry ≥16 atmospheres vs. ≥18 atmospheres in ABSORB trials). It is uncertain if these differences represent a change in clinical outcomes. 

### 4.1. One-Year Follow-up Data

**Figure 1:**
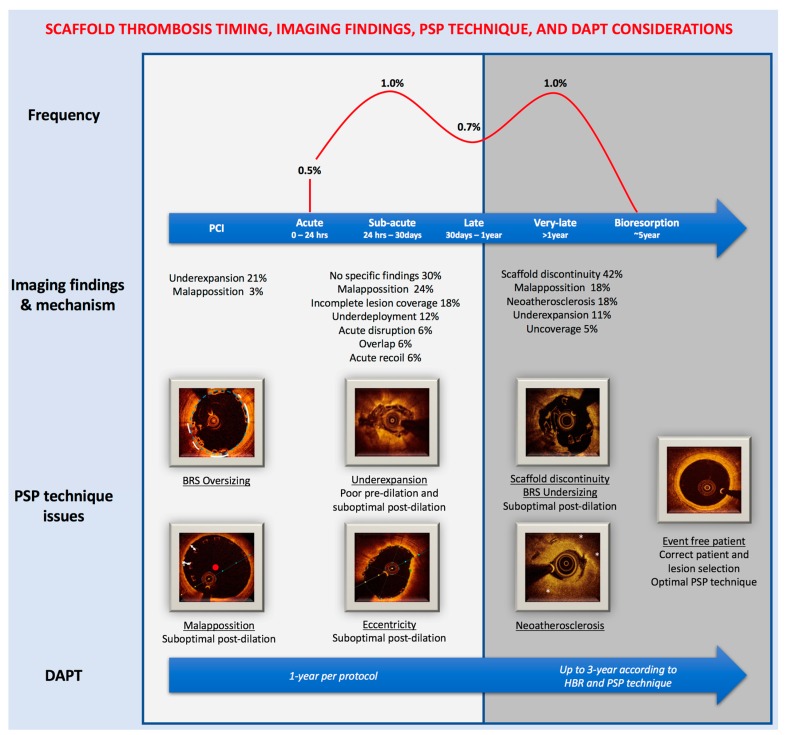
Scaffold thrombosis and PSP technique. The frequency of scaffold thrombosis according to the meta-analysis of Collet et al. [27] Imaging findings and mechanism according to Tanaka et al. [9], Sotomi et al. [28] and Yamaji et al. [29] Mechanism of failure as defined in Table 2. In the PSP technique issues, the primary failure mechanism is underlined followed with the suboptimal technique related. DAPT: Dual-antiplatelet therapy; HBR: High-bleeding risk.

**Figure 2:**
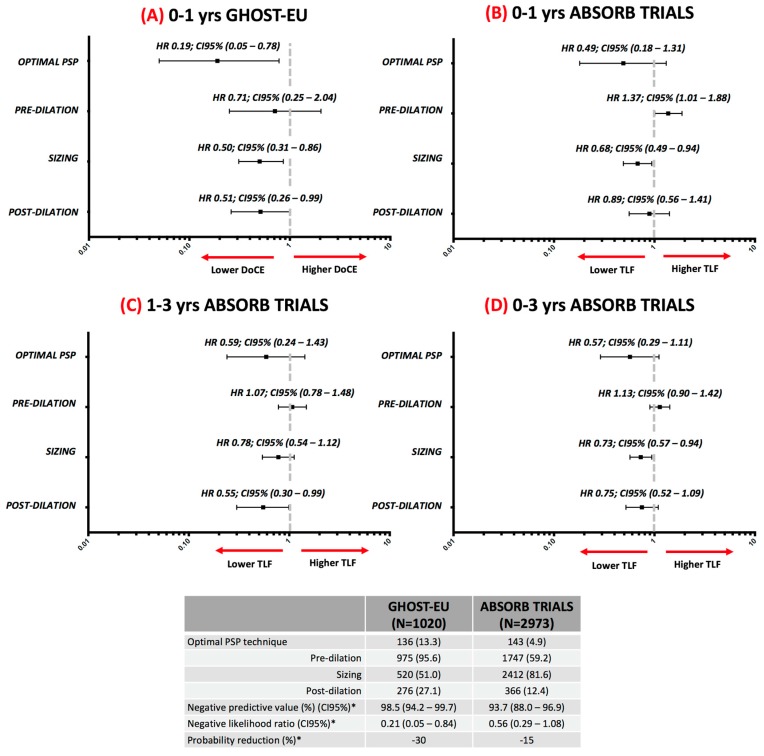
Impact of PSP technique on device-oriented composite endpoint following bioresorbable scaffolds implantation. HR and 95% CI values of the GHOST-EU trial were adapted from [8] and of the ABSORB trials were adapted from [10]. * Predictive values and likelihood ratio for GHOST-EU is calculated at 1-year and for ABSORB trials at 3-years. DoCE and TLF are defined in Table 2. HR: Hazzard ratio; CI: Confidence Interval; DoCE: device-oriented composite endpoint; TLF: Target Lesion Failure.

During the following year after implantation the process of arterial healing and reendothelization takes place, within this period, it is appeared to be the first peak of adverse events. (Figure 1) [14]. Data from the two commented studies showed that in this period a correct vessel sizing according to the RVD is the most critical determinant of event-free rate (Figure 2A,B), with a relative reduction of the hazard risk between 32% and 61%. These findings support the previous results of a post-hoc analysis of the ABSORB III trial in which the treatment of very-small vessel (QCA-RVD <2.25 mm) was a strongly related to adverse events [10]. In addition, it matches the findings of the MICAT registry in which a low post-PCI MLD by QCA was a predictor of scaffold thrombosis [7]. The most consistent finding related to an adverse event during the first year is the implantation of an oversized BRS (Table 1, scaffold sizing) in a small vessel which leads to a high scaffold footprint, disturbance on shear stress distribution, and platelet activation. Eventually, there are also some reports that suggest the relationship of an optimal post-dilation and the event-free rate in the acute and late period [13].

### 4.2. Three-Year Follow-Up Data

Between the first and third year, the process of bioresorption takes place and this appeared to be a second peak of adverse events (Figure 1) [14]. The intraluminal scaffold dismantling is a specific mechanism of failure of BRS, which consists in the translocation of scaffold elements into the lumen during the bulk erosion process between years 1 and 3 [30]. Data from the ABSORB trials showed that in this period a correct vessel sizing has no relationship with adverse events. Whereas an optimal post-dilation was the most important determinant of event-free rate (Figure 2C), with a relative reduction of the hazard risk of 45% [10]. This finding reinforces the importance of the systematically post-dilation, but also the use of NC slightly oversized according to the RVD or scaffold diameter, up to 0.5 mm to avoid the scaffold over-expansion, and a dilation pressure of ≥18 atmospheres. The mechanism by which an optimal post-dilation can prevent very-late events could be: maximize scaffold dimensions, embed struts into plaque, avoid acute malapposition, and reduce shear stress [10]. Many of these mechanisms have been observed in intravascular imaging studies of very-late scaffold thrombosis cases [29]. Meanwhile, the MICAT authors, have suggested that the very-late events could also be related to a suboptimal sizing of the BRS and vessel. During the 1 to 3-year period, they found that BRS undersizing (Table 1, scaffold sizing) was strongly related to the adverse event rate [11]. Taking into account all these findings, the very-late events appear to be related to different mechanisms than the acute/late events but also associated with a suboptimal PSP technique.

### 4.3. Evidence in Context

We consider that it rational to accept that an optimal PSP technique is related to a lower rate of adverse outcomes. However, it should not be forgotten that these data also have several limitations, Table 2 lists some of them. Patient and lesion selection are also key factors to take into account to achieve optimal clinical outcomes in the patients treated with BRS [31]. Eventually, it is important to highlight that all these data are derived from post-hoc analysis, so in the best of the scenarios, these results are hypotheses to be tested. Unfortunately, due to methodological and ethical issues, this seems to be unfeasible. Finally, the percentage of patients treated with an optimal PSP technique was low in both studies (Figure 2), making it difficult to analyze the result due to the very low rate of events in this specific sub-group. 

## 5. DAPT Regimen and PSP Technique

There are few studies addressing a specific DAPT regimen in patients treated with BRS [13,32]. Actually, most of the actual recommendations are based on expert consensus [14,15]. A full review of the recommended DAPT regimens is out of the scope of this report. Briefly, within the context of the ABSORB trials, the protocol specifies 12-months of DAPT regardless of the treatment indication [33]. Meanwhile, expert recommendations and ESC guidelines state a similar approach of ≥12-months (Class IIa C) with the possibility of prolonging it up to 30-month, regardless of the treatment indication [14,15]. Taking into account the currently recommended DAPT regimen and the risk of device thrombosis, compliance with DAPT and bleeding risk should be evaluated before BRS implantation.

As many studies have shown that BRS have a higher rate of device thrombosis when compared with second-generation DES, with a strong relationship with a suboptimal PSP technique and DAPT discontinuation (Figure 2) [6,7,11,13]. In this context, the prolongation of DAPT up to the time of complete bioresorption could be an option to reduce the risk of scaffold thrombosis. However, whether we should prolong it to all patients or to those at higher risk of an event is uncertain, given the bleeding risk associated. 

**Figure 3:**
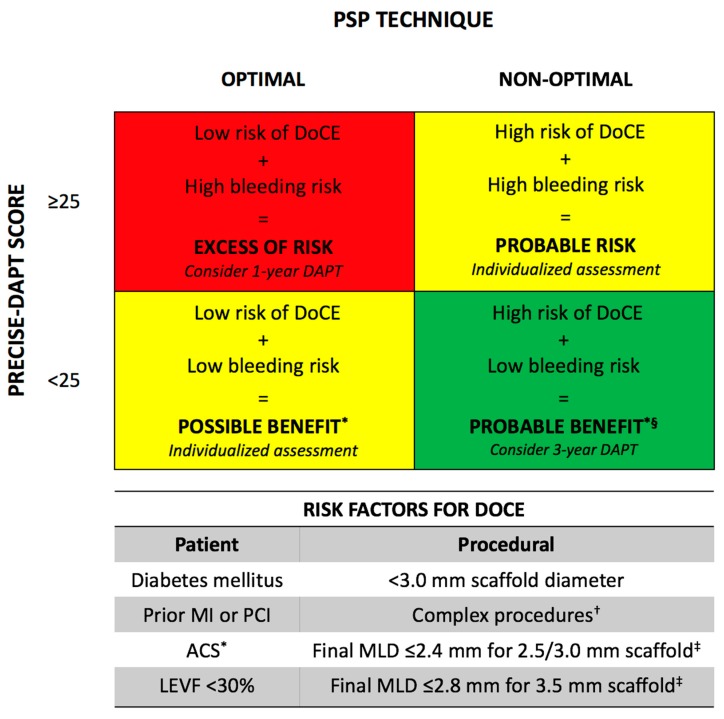
Risk factors to be considered for deciding to whom prolong dual-antiplatelet therapy following bioresorbable scaffold implantation. * Prasugrel and ticagrelor should be preferred over clopidogrel in ACS. † Complex procedures include ACC/AHA B2/C type lesions, >1 BRS implanted, or any other unfavorable clinical, angiographic, and procedural characteristics. ‡ Assessed by quantitative coronary angiography after PCI.4 § More intense regiment of DAPT could be considered in stable coronary artery disease patients: prasugrel/ticagrelor plus aspirin for 1-month, then de-escalation to clopidogrel plus aspirin [14]. DAPT: dual antiplatelet therapy; DoCE: device-oriented composite endpoint; MI: myocardial infarction; PCI: percutaneous coronary intervention; ACS: acute coronary syndrome; LEVF: left ejection ventricular fraction; MLD: minimum lumen diameter.

The PRECISE-DAPT score is a prediction model of the out-of-hospital bleeding risk during DAPT, although its derivation cohort was composed only of metallic stents, this score could be useful in the BRS setting [34]. An optimal PSP technique, assessed by the PSP score, has been related to a very-high negative predictive value (98.5%) at 1-year scaffold thrombosis [8] and also very-high negative predictive value (93.7%) at 3-year [10]. Both score could be applied together to weight the ischemic/bleeding risk of each patient to select those in whom prolonged DAPT could be safe and effective (Figure 3).

In those patients at low-risk of bleeding/high-risk of ischemic events (Figure 3, green panel), prolonged DAPT appears rational and safe/effective. Conversely, prolonged DAPT in those patients at high-risk of bleeding/low-risk of ischemic events (Figure 3, red panel) may appear unsafe and ineffective. Patients at intermediate-risk (Figure 3, yellow panels) should undergo an individualized risk/benefit assessment to tailor the DAPT duration between 1 to 3-year. In all patients, risk factors for DoCE should be considered, while bleeding risk and events should be periodically assessed [7,14].

Currently, the BVS LATE study will evaluate the optimal duration of antiplatelet therapy after BRS implantation to reduce late coronary arterial thrombotic events (NCT02939872).

## 6. Future Perspective 

The 30-day endpoint of the ongoing ABSORB IV trial (NCT02173379) was recently presented [35]. A total of 2604 patients were randomized (1:1 ratio) to Absorb BRS vs. second-generation DES, the primary endpoints are TLF at 30 days and TLF between 3 and 7–10 years (pooled with ABSORB III). At 30-day, the TLF rate in patients treated with BRS was non-inferior when compared to DES (5.0% vs. 3.7%, *p*_NI_ = 0.02). There was a trend towards a higher rate of device thrombosis in the patient treated with BRS when compared to DES (0.6% vs. 0.2%; HR 4.05, CI 95% (0.86–19.07); *p* = 0.06). Regarding the PSP technique (defined as in the ABSORB trials, Table 2), optimal pre-dilation was performed in 15.2%, correct sizing 96.3% (2.5% of the included patients have a vessel of <2.25 mm), and optimal post-dilation in 15.2% of the cases. In this context, the authors concluded that despite the trial was successful in eliminating the very-small vessels; an optimal PSP technique rate was still low in BRS patients (8%). Interestingly, DES outcomes were also improved with an optimal PSP technique, so operators should be encouraged to apply this technique with DES as well. The second co-primary endpoint should be completed in April 2024.

Despite the safety issues that led this first-generation device to be discontinued, there are still several BRS devices in clinical and pre-clinical studies; this indicates a positive future for this promising technology [36]. Specifically, the Magmaris BRS (Biotronik), has initiated the MAGSTEMI trial (NCT03234348), which will be the first randomized clinical trial with this device. However, due to differences in the composition of the scaffold (BVS: poly-L-lactic acid vs. Magmaris: magnesium alloy), it is unknown if the same PSP technique definitions could be useful.

Abbott Vascular is working on the next generation device, Falcon BRS, some of the possible characteristics of this new device are listed in the Table 3 [37]. However, this device has not been used in humans, and it is unknown when it will be available for clinical use.

## 7. Conclusions

Despite the excellent performance of newer-generation DES, patients are still at long-term high risk of device-related events; this fact is the rationale that should continue pushing our efforts on the development of this technology. However, lessons learned from this first-generation device should be the cornerstone in the pursuit of patient wellness. A correct patient and lesion selection, application of an optimal implantation technique, intravascular imaging guidance, specific DAPT regimen, and improvement of the device limitations will be essential to determining the destiny of this technology.

## Figures and Tables

**Table 1:** Comparison of angiography—QCA and intravascular imaging-guided PSP techniques.

PSP Step	Angiography—QCA Guided	Intravascular Imaging Guided
Pre-dilation	• To assess the calcification of the lesion. • To confirm the full expansion of the dilation balloon. • To check that <30% of diameter stenosis is obtained.	To assess plaque composition [19]: • Fibrous plaque • Calcified plaque • Lipid-rich plaque • Thin-cap fibrous atheroma To determine the diameter stenosis after pre-dilation.
Scaffold sizing	• To estimate the RVD assessed by online QCA. Depending on the RVD the scaffold diameter is choose. * If the proximal and distal RVD differed, the mean value is used. • The pre-dilation balloon could be used to estimate the RVD. • To rule-out BRS oversizing (ratio of BRS nominal diameter to RVD >1.15) [11]. • To rule-out BRS undersizing (ratio of BRS nominal diameter to RVD <0.85) [11]. • To avoid scaffold mismatch.	• To estimate the mean RVD and choose the scaffold diameter. Depending on the RVD the scaffold diameter is choose. * • To determine the landing zone and scaffold length
Post-dilation	• To select a balloon >1:1 ratio with RVD up to 0.5 mm. • To confirm the full expansion of the dilation balloon. • To check that <10% of diameter stenosis is obtained. • To optimized overlap zone [20]. • To determine post-PCI MLD assessed by online QCA. ○ ≥2.4 mm for 2.5/3.0 mm BRS ○ ≥2.8 mm for 3.5 mm BRS	The following should be assessed: • Underexpasion: if in-scaffold area stenosis is >20% or MLA <4.0 mm^2^ [21]. • Expansion asymmetry: assessed by the eccentricity index (minimum and maximum scaffold/stent diameter per cross section <0.7) [22]. • Malappostion: incomplete scaffold apposition >300 µm with a longitudinal extension >1.0 mm [23] • Intra-scaffold mass: diameter >500 µm with longitudinal extension >3.0 mm [24].

* Implantation of a 2.5 mm diameter scaffold in a vessel with a proximal/distal RVD ≥2.5 mm and <2.75 mm; 3.0 mm diameter scaffold in a vessel with a proximal/distal RVD ≥2.75 mm and <3.25 mm; or 3.5 mm diameter scaffold in a vessel with a proximal/distal RVD ≥3.25 mm and ≤3.75 mm. RVD: reference vessel diameter; QCA: quantitative coronary angiography; PCI: percutaneous cardiac intervention; MLD: minimal lumen diameter; MLA: minimal lumen area; BRS: bioresorbable scaffolds.

**Table 2:** Comparison of the study population, methodology, and definition of the optimal PSP technique in the GHOST-EU registry and the ABSORB TRIALS.

Trial Characteristic	GHOST-EU Registry	ABSORB TRIALS
Studies designs (publication date)	Retrospective registry of consecutive cases (February 2015)	ABSORB II RCT (January 2015), ABSORB III RCT (November 2015), ABSORB CHINA RCT (December 2015), ABSORB JAPAN RCT (December 2015), ABSORB EXTEND registry (April 2015)
Post-hoc analysis	Yes	Yes
Patients	1020	2973
Clinical settings	CAD, ACS (including STEMI), CTO, Ostial, Bifurcations, LMCA and ISR.	CAD and ACS
Scaffold overlap	Yes	Only ABSORB II and EXTEND
Lesion characteristics	No lesion length restriction Up to four lesions	De novo Lesion length <28 mm (except ABSORB II) Up to two lesions
Intravascular imaging	Not mandatory, performed in a minority	Not mandatory, performed in a minority
Endpoint	DoCE: Cardiac death, target-vessel myocardial infarction, or clinically-driven target lesion revascularization	TLF: Cardiac death, target-vessel myocardial infarction, or ischemia-driven target lesion revascularization
Core lab analysis and event adjudication	No	Yes (different from each study)
Follow-up	Up to 1-year	Up to 3-year
Optimal PSP technique	All steps performed correctly in all lesions. Angiography guided. Offline QCA analysis.	All steps performed correctly in all lesions. Angiography guided. Offline QCA analysis.
Pre-dilation	NC balloon ≥1:1 ratio with RVD	NC balloon ≥1:1 ratio with RVD
Sizing	According to manufacturer recommendations *	QCA-RVD ≥2.25 mm and ≤3.75 mm
Post-dilation	NC balloon >1:1 ratio with RVD up to 0.5 mm at ≥16 atmosphere	NC balloon at ≥18 atm and with nominal diameter larger than the nominal scaffold diameter, but not >0.5 mm larger

* Same as in Table 1. RVD: reference vessel diameter; QCA: quantitative coronary angiography; RCT: Randomized Clinical Trial; CAD: Coronary Artery Disease; ACS: Acute Coronary Syndrome; STEMI: ST-segment Elevation Myocardial Infarction; CTO: Chronic Total Occlusion; LMCA: Left Main Coronary Artery; ISR: In-stent restenosis; DoCE: device-oriented composite endpoint; TLF: Target Lesion Failure; NC: non-compliant.

**Table 3:** Falcon Bioresorbable Scaffolds Design and Development Plans.

Improvement	Comment [37]
Strut thickness	Reduction from 157 to 99 µm May reduce acute thrombogenicity and achieve full endothelialization earlier Increase deliverability
Increase size matrix	From 14 to 40 sizes Longer scaffold to avoid overlap New diameters for optimal sizing
Delivery balloon system	Reduce compliance of the delivery balloon Optimized for PSP technique More accurate deployment diameters
Intravascular imaging	Optical Coherence Tomography guidance to ensure optimal implantation
Radial strength resorption	Will maintain poly-l-lactic acid structure Will maintain poly-dl-lactic acid/Everolimus Gradual loss of radial strength after complete coverage
